# Supplementary material for: A screening system for identifying interacting proteins using biomolecular fluorescence complementation and transposon gene trap
Source: PLoS One. 2021 May 14;16(5):e0251240. doi: 10.1371/journal.pone.0251240 (PMC8121353; doi:10.1371/journal.pone.0251240)
Supplement: S1 File — (PDF) [file pone.0251240.s001.pdf]

S1 Appendix. Materials

The nucleotide and amino acid sequences of p65-mKGN and p50-mKGC.

(1) p65-mKGN

p65 (partial fragment): **lower case (bold)**

linker: lower case (underline)

mKGN: UPPER CASE

Nucleotide sequence

5′-**atgaacactgccgagctcaagatctgccgagtgaaccgaaactctggcagctgcctcggtggggat**
gagatcttcctactgtgtgacaaggtgcagaaagaggacattgaggtgtatttcacgggaccaggctgg
gaggcccgaggctccttttcgcaagctgatgtgcaccgacaagtggccattgtgttccggacccctccc
tacgcagaccccagcctgcaggctcctgtgcgtgtctccatgcagctgcggcggccttccgaccgggag
ctcagtgagcccatggaattccagtacctgccagatggagcggccgccaattccgctgacggcggcgga
ggatcgggtggtagtggtggttcaggaggaggatcgacccaaggaaccggtATGGTGAGCGTGATCAAG
CCCGAGATGAAGATGAGGTACTACATGGACGGCTCCGTCAATGGGCATGAGTTCACAATCGAGGGTGAG
GGCACAGGCAGACCTTACGAGGGACATCAGGAGATGACACTGCGCGTCACAATGGCCGAGGGCGGGCCA
ATGCCTTTCGCCTTCGACCTGGTGTCCCACGTGTTCGCCTACGGCCACAGGGTGTTTACCAAGTACCCA
GAAGAGATCCCAGACTATTTCAAGCAGGCCTTTCCTGAGGGCCTGTCCTGGGAGAGGTCCCTGGAGTTC
GAGGACGGCGGCTCCGCCTCCGTGAGCGCCCACATCAGCCTGAGGGGCAACACCTTCTACCACAAGTCC
AAGTTCACCGGCGTGAACTTCCCCGCCGACGGCCCCATCATGCAGAACCAGAGCTACGACTGGGAGCCC
TCCGAGGAGAAGATCACCGCCAGCGACGGCGTGCTGAAGGGCGACGTGACCATGTACCTGAAGCTGGAG
GGCTAA-3′

Amino acid sequence

mntaelkicrvnrnsgsclggdeifllcdkvqkedievyftgpgweargsfsqadvhrqvaivfr
tppyadpslqapvrvsmqlrrpsdrelsepmefqylpdgaaansadggggsggsggsgggstqgt
gMVSVIKPEMKMRYYMDGSVNGHEFTIEGEGTGRPYEGHQEMTLRVTMAEGGPMPFAFDLVSHVF
AYGHRVFTKYPEEIPDYFKQAFPEGLSWERSLEFEDGGSASVSAHISLRGNTFYHKSKFTGVNFP
ADGPIMQNQSYDWEPSEEKITASDGVLKGDVTMYLKLEG*

(2) p50-mKGC

p50 (partial fragment): **lower case (bold)**

linker: lower case (underline)

mKGC: UPPER CASE

Nucleotide sequence

5′-**atgaatgcatccaacttgaaaattgtaagaatggacaggacagctggatgtgtgactggaggggag gaaatttatcttctttgtgacaaagttcagaaagatgacatccagattcgattttatgaagaggaagaa aatggtggagtctgggaaggatttggagatttttcccccacagatgttcatagacaatttgccattgtc ttcaaaactccaaagtataaagatattaatattacaaaaccagcctctgtgtttgtccagcttcggagg aaatctgacttggaaactagtgaaccaaaacctttcctctactatcctgga**gcggccgccaattccgct gacggcggcggaggatcgggtggtagtggtggttcaggaggaggatcgacccaaggaaccggtATGGGC GGCAACCACAAGTGCCAGTTCAAGACCACCTACAAGGCCGCCAAGGAGATCCTGGAGATGCCCGGCGAC CACTACATCAGCCACAGGCTGGTGAGGAAGACCGAGGGCAACATCACCGAGCTGGTGGAGGACGCCGTG GCCCACTCCTAA-3′

Amino acid sequence

mnasnlkivrmdrtagcvtggeeiyllcdkvqkddiqirfyeeeenggvwegfgdfsptdvhrqf aivfktpkykdinitkpasvfvqlrrksdletsepkpflyypgaaansadggggsggsggsgggs tqgtgMGGNHKCQFKTTYKAAKEILEMPGDHYISHRLVRKTEGNITELVEDAVAHS*

S1 Table. Primers or restriction enzyme to obtain fragments for in-fusion reaction.

Gene or vector	Template	Primer or restriction enzyme
Common for *mKGC-CACYBP*, *mKGC-HSP90AB1*, *mKGC-PKM*, *mKGC-KRT8*	cDNA from Each clone using manufacture's oligo dT adaptor primer	5′-CCACCGGTAATTCCGCTGACGGC-3′ (anneals to the linker)
		5′-CTGATCTAGAGGTACCGGATCC-3′ (anneals to t he adaptor)
Subcloning vector for *mKGC-geneX*	pPB-P$_{CMV}$-*mKGC*	5′-GTCAGCGGAATTACCGGTGG-3′
		5′-GTACCTCTAGATCAGGTACCGGAACTGCAGCAGAGAATTC-3′
Common primers for transferring *mKGC-geneX* from the subcloning vector to the expression vector at *Bam*HI site	Each subcloning vector	5′-CGCTAGCATTGGATCCCACCATGGGCGGCAACCAC-3′
		5′-TAGAACTAGTGGATCTGCTGCACTGATCTAGAGGTACCGGATC-3′

Expression vector for *mKGC-geneX*	pPB-P_{CMV}-*mKGC*-IRES-$Puro^R$	*Bam*HI
HSPA8-mKGC	cDNA from clone #7	5′-AAGCTTGGATCAGCGACCATGTCCAAGGGACCTGCAGTTGGTATTG-3′
		5′-GGTCTTGAACTGGCACTTGTGGTTG-3′
ANXA2-mKGC	cDNA from clone #6	5′-AAGCTTGGATCAGCGAGCTTCCTTCAAAATGTCTACTGTTCACGAAATCCTG-3′
		5′-GGTCTTGAACTGGCACTTGTGGTTG-3′
Expression vector for *geneX-mKGC*	pPB-P_{CMV}-mKGC-IRES-$Puro^R$	5′-TGCCAGTTCAAGACCACCTACAAG-3′
		5′-CGCTGATCCAAGCTTTGTTC-3′

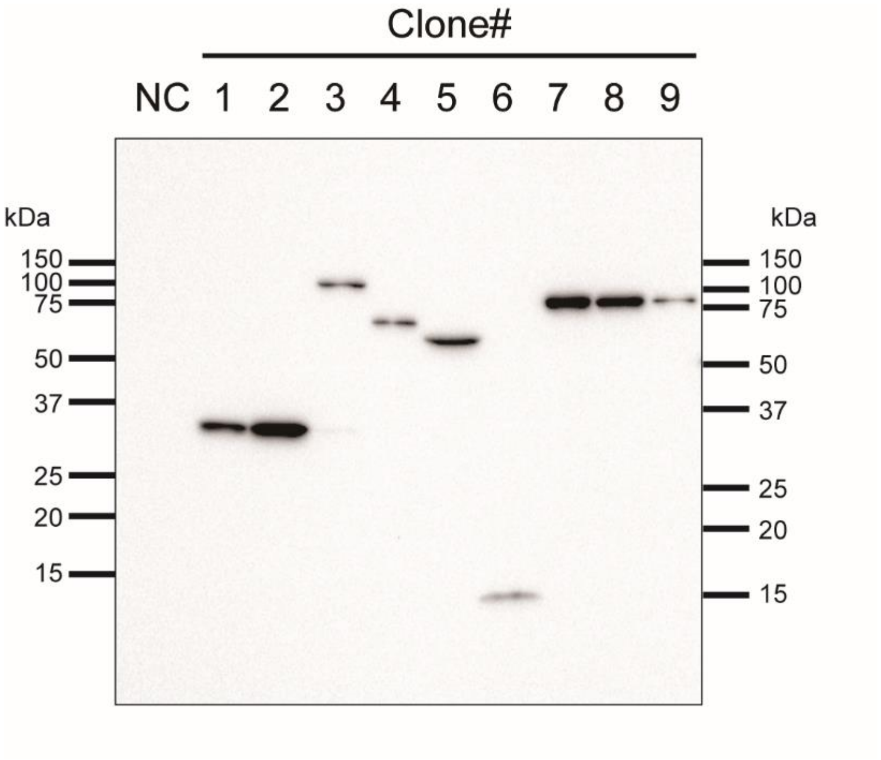

S1 Fig. Confirming expression of mKGC fusion protein.
The protein lysate extracted from each clone was subjected to SDS-PAGE, and immunobloting using an anti-mKGC antibody was performed.

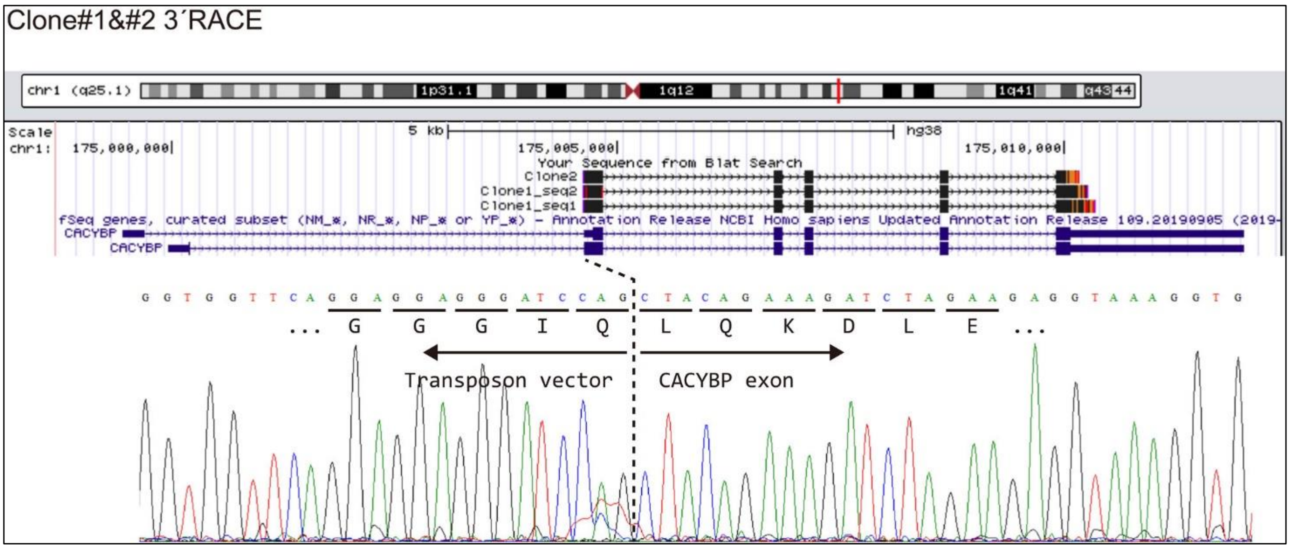

Clone#3 3´RACE

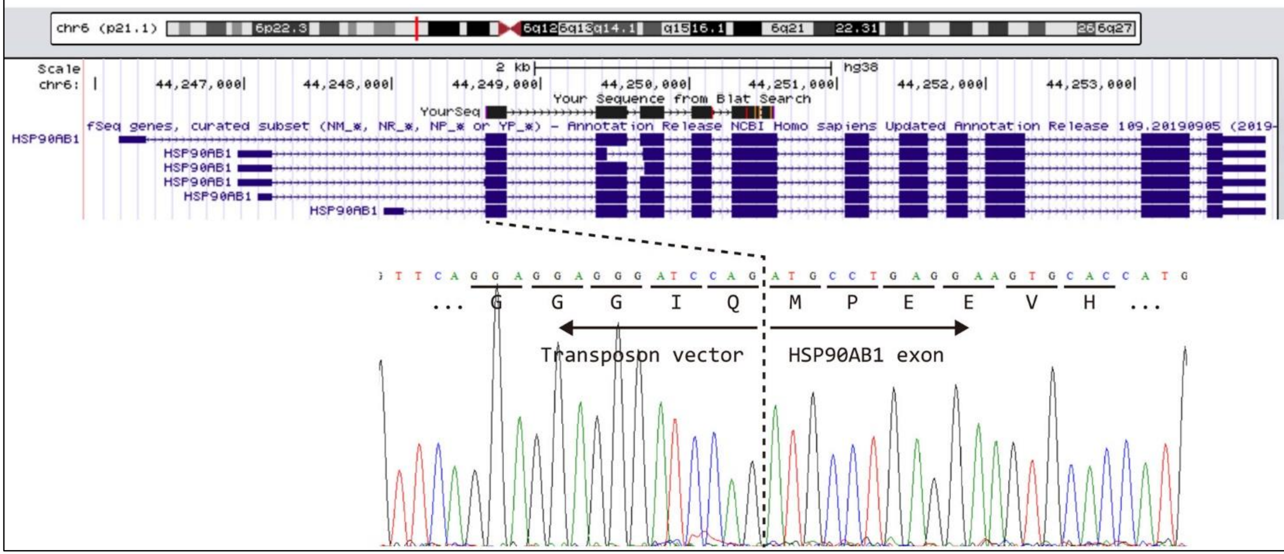

Clone#4 3´RACE

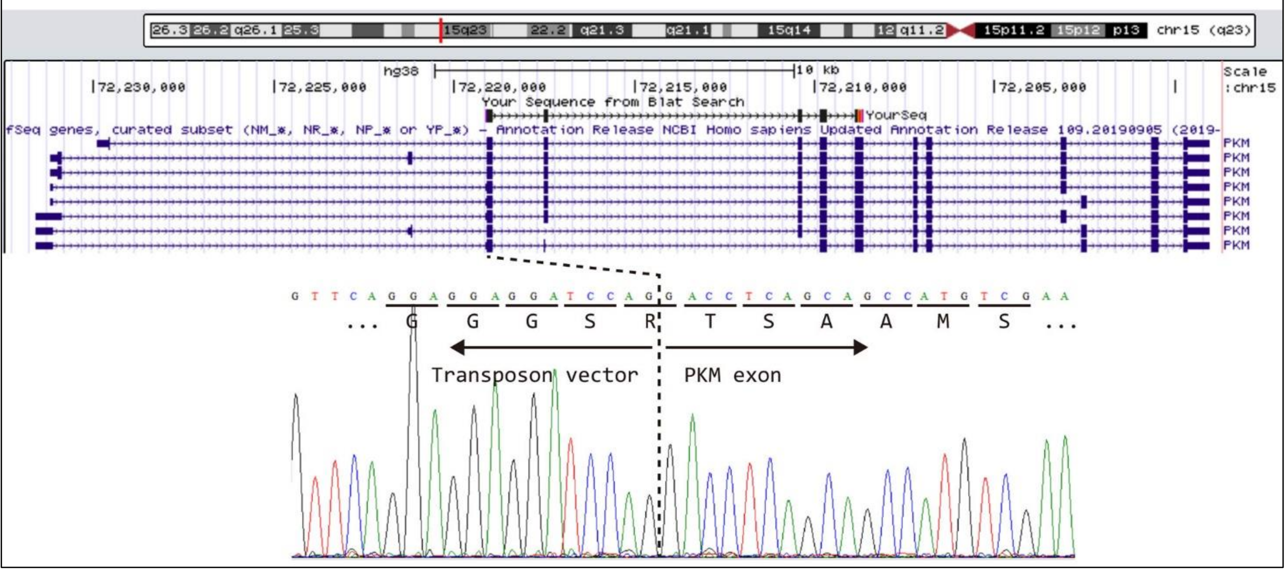

Clone#5 3´RACE

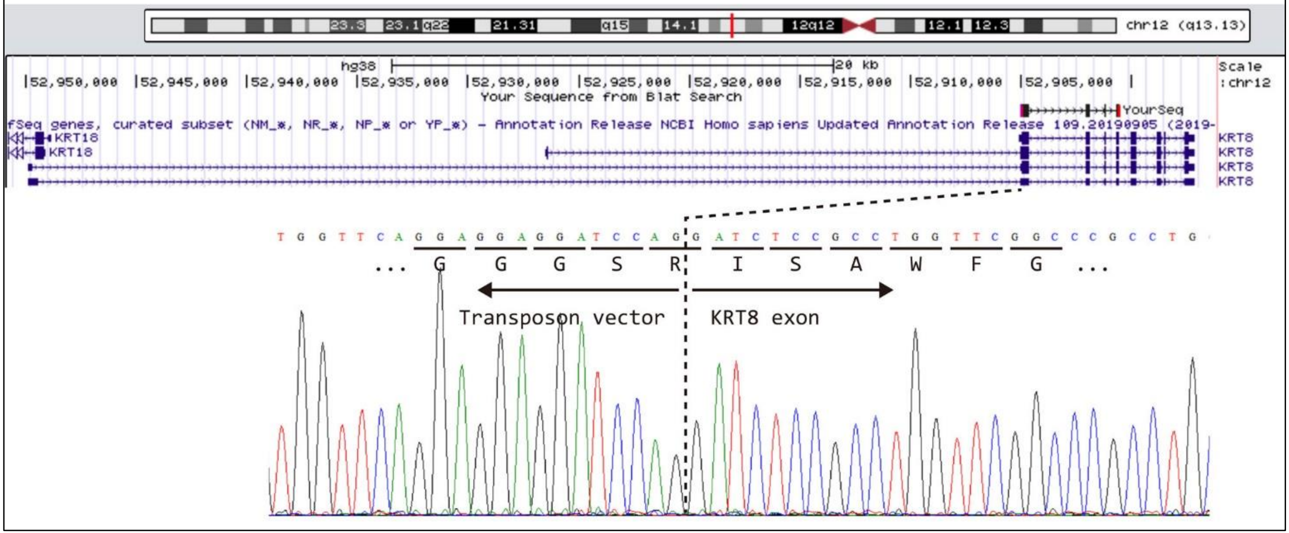

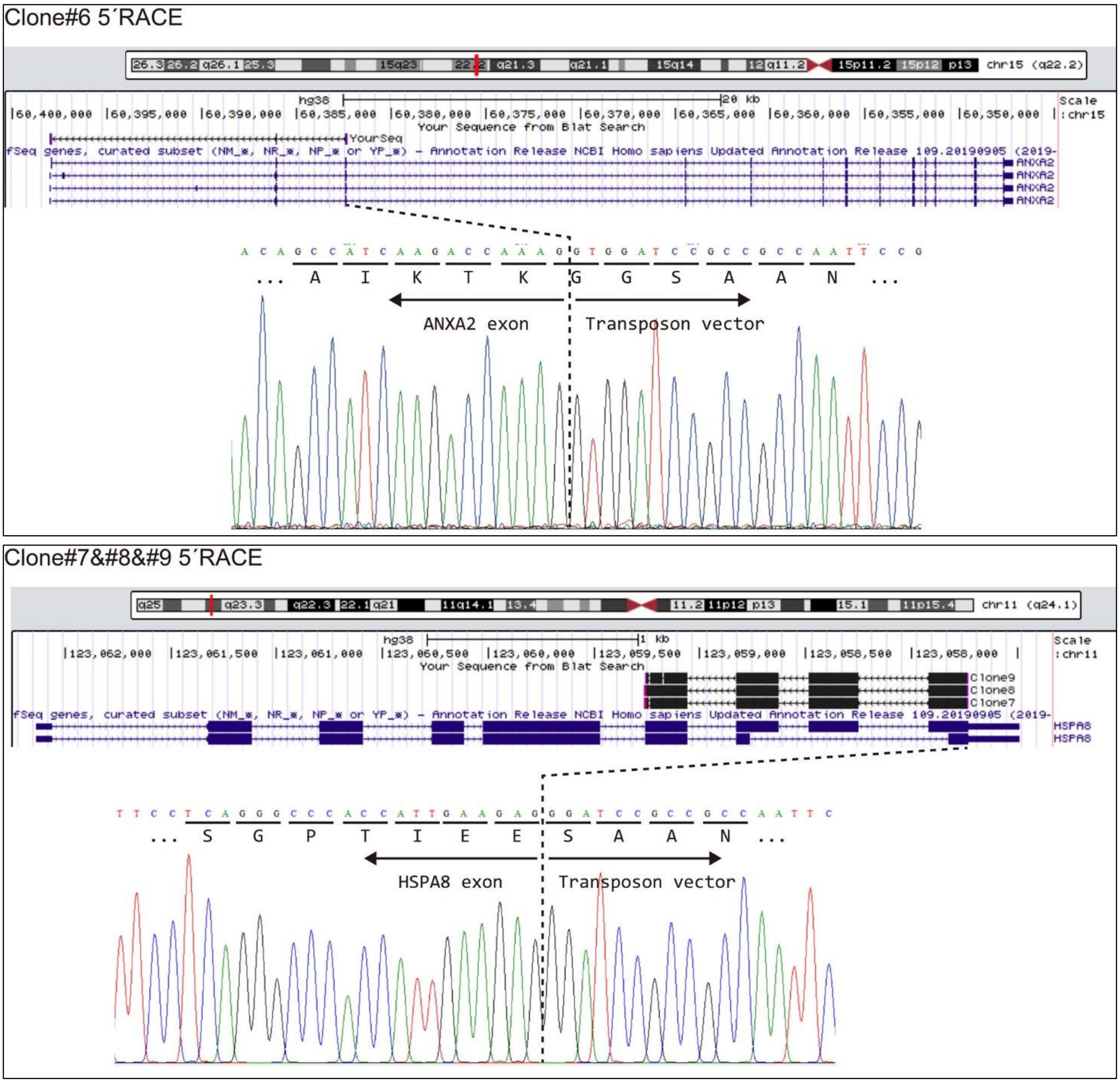

S2 Fig. 5′- or 3′-RACE analyses to identify genes fused to mKGC.

A coding sequence fused to mKGC was obtained by RACE and analyzed using the BLAT tool (https://genome.ucsc.edu/cgi-bin/hgBlat). In each panel, the sequence match on the UCSC browser is shown above, and the schematic diagram below shows how the identified gene exon is linked to the coding sequence on the vector inserted into the genome. Note that, in clones #1 and #2 (these were derived from independent screenings), mKGC was linked to the same exon of *CACYBP* on the cDNA, and the vector insertion sites were identical on the genome. In clones #7, 8 and 9 (these were also derived from independent screenings), mKGC was linked to the same exon of *HSPA8* on the cDNA, while the vector insertion sites on the genome were different as revealed by the sequence analysis of the genomic DNA.

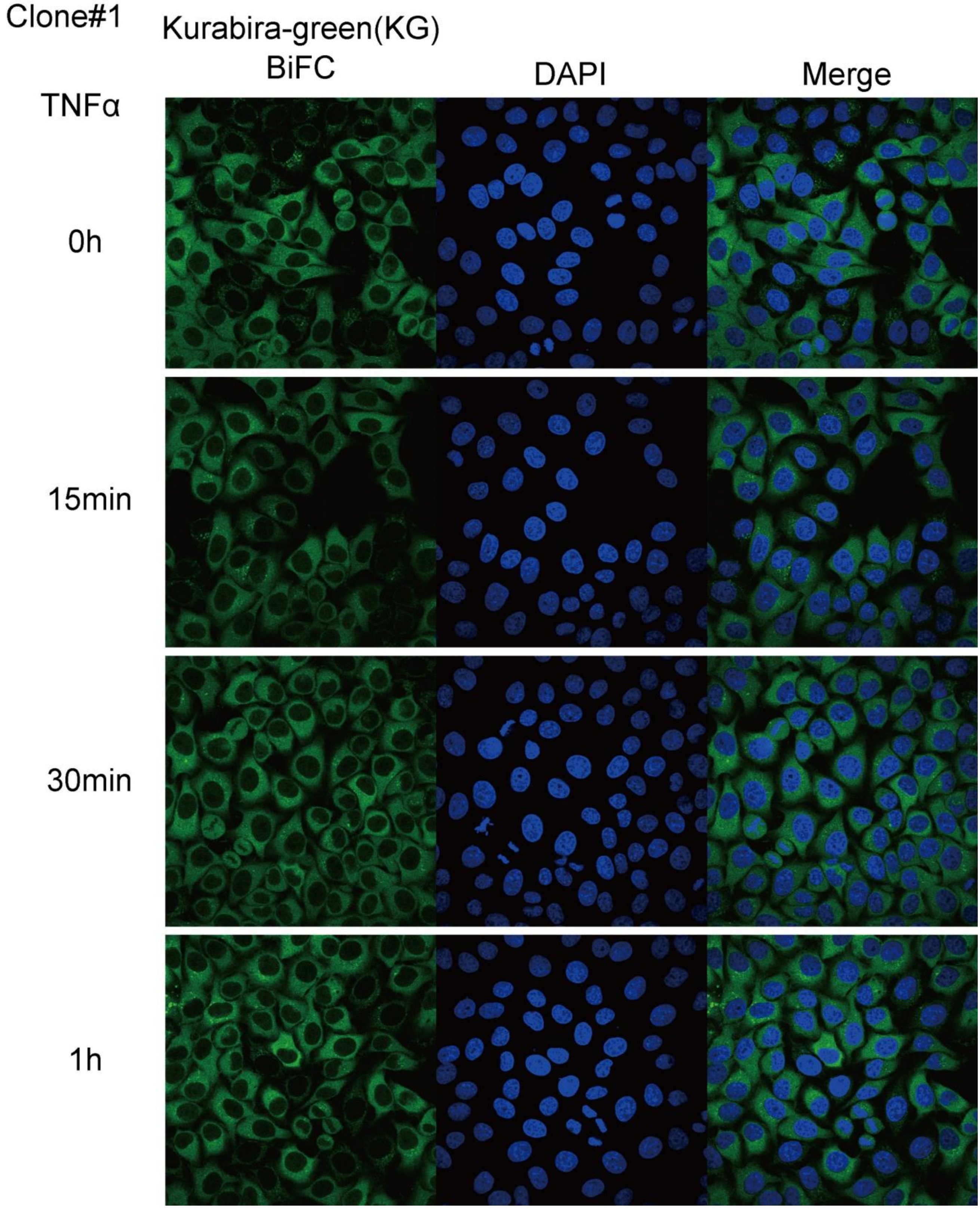

Clone#1
Kurabira-green(KG)
BiFC
DAPI
Merge
TNFα
0h
15min
30min
1h

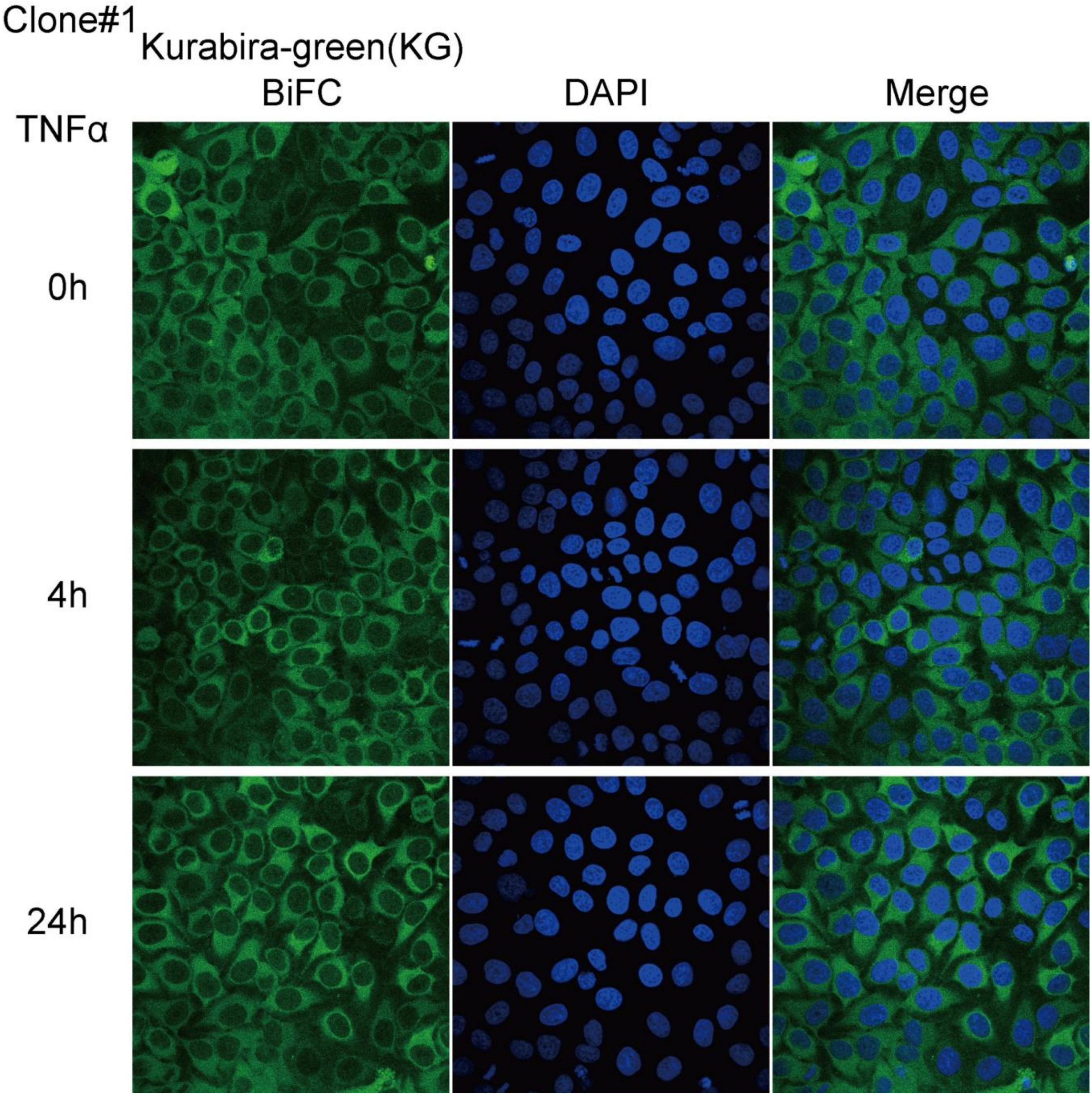

S3 Fig. Confocal microscopic analysis of clone #1 upon stimulation by TNFα.

After Stimulation of clone#1 (bait: p65-mKGN), in which mKGC-CACYBP was identified, with 20 ng/mL of TNFα for the time indicated, 3.8% formalin fixation and DAPI staining was performed, and analyzed with a confocal microscope. The BiFC signal was observed specifically in the cytoplasmic region, and small speckle-like aggregates were observed in cytoplasmic regions. This pattern did not seem to change during the 0–24h period of TNFα stimulation.
